# Triatomine fauna in the state of Bahia, Brazil: What changed after 40 years of the vector-control program?

**DOI:** 10.1590/0037-8682-0732-2021

**Published:** 2022-07-25

**Authors:** Gilmar Ribeiro-Jr, Renato Freitas de Araújo, Cristiane Medeiros Moraes de Carvalho, Gabriel Muricy Cunha, Fernanda Cardoso Lanza, Diego Lopes Paim Miranda, Orlando Marcos Farias de Sousa, Carlos Gustavo Silva dos Santos, Eduardo Oyama Lins Fonseca, Roberto Fonseca dos Santos, Renato Barbosa Reis, Rodrigo Gurgel-Gonçalves, Mitermayer Galvão Reis

**Affiliations:** 1 Fundação Oswaldo Cruz, Instituto Gonçalo Moniz, Salvador, BA, Brasil.; 2 Secretaria de Saúde do Estado da Bahia, Centro de Saúde José Maria de Magalhães Neto, Salvador, BA, Brasil.; 3 Universidade Federal da Bahia, Faculdade de Medicina da Bahia, Salvador, Brasil.; 4 Ministério da Saúde, Secretaria de Vigilância em Saúde, Coordenação Geral de Vigilância de Zoonoses e Doenças de Transmissão Vetorial, Brasília, DF, Brasil.; 5 SENAI, Instituto de Inovação em Sistemas Avançados de Saúde, ISI/SENAI CIMATEC, Salvador, BA, Brasil.; 6 Laboratório Central de Saúde Pública Professor Gonçalo Moniz, Salvador, BA, Brasil.; 7 Universidade Salvador, Salvador, BA, Brasil.; 8 Universidade de Brasília, Faculdade de Medicina, Laboratório de Parasitologia Médica e Biologia de Vetores, Brasília, DF, Brasil.; 9 Yale University, Connecticut, USA.

**Keywords:** Triatominae, Northeastern Brazil, Trypanosoma cruzi, Chagas disease, Public health

## Abstract

**Background:**

Neglected tropical diseases are a growing threat to global health, and endemic Chagas disease has emerged as one of the most important health problems in America. The main strategy to prevent *Trypanosoma cruzi* transmission is chemical control of vectors. This study presents a descriptive analysis of synanthropic triatomines before and after the implementation of a vector-control program in Bahia, Brazil.

**Methods:**

Descriptive analysis and geospatial statistics were performed on triatomine data, (1) the relative abundance and (2) proportional spatial distribution, from Bahia during two periods: (A) 1957 to 1971 and (B) 2006 to 2019.

**Results:**

We observed a decrease in the relative abundance of *Panstrongylus megistus* (A: n=22.032, 61.9%; B: n=1.842, 1.0%) and *Triatoma infestans* (A: n=1.310, 3.7%; B: n=763, 0.43%), as well as an increase in the relative abundance of *T. sordida* (A: n=8.314, 23.4%, B: n=146.901, 81.6%) and *T. pseudomaculata* (A: n=894, 2.5%, B: n=16.717, 9.3%).

**Conclusions:**

Our results indicate a clear reduction in the occurrence of *P. megistus* and *T. infestans* (last record in 2015) and an increase in the relative abundance and geographical distribution of *T. sordida* and *T. pseudomaculata* after 40 years of the vector-control program. The high frequency of other triatomine species in the municipalities of the state of Bahia and their abundance in recent years highlight the need to reinforce permanent entomological surveillance actions to prevent Chagas disease.

## INTRODUCTION

Triatomines are blood-sucking insects (Hemiptera: Reduviidae) that are vectors of the parasite *Trypanosoma cruzi* (Chagas, 1909), the etiological agent of Chagas disease, also known as American trypanosomiasis. Some synanthropic triatomine species adapt to anthropic changes within their natural landscape, colonizing the household environment (animal breeding sites) and occasionally inside houses, increasing the risk of *T. cruzi* transmission^1^ ^,^ ^2^. Many triatomines invade houses, but few can initiate the processes of colonization and domiciliation, which depend on the characteristics of the invasive species^3^ ^,^ ^4^, the invaded dwelling^5^ ^,^ ^6^ ^,^ and the environment around the household^7^.

In Brazil, some triatomine species have succeeded in occupying the domestic environment and expanding their spatial distribution beyond their original biomes^8^ through active and passive dispersal^9^ ^,^ ^10^. One of these species is *Triatoma infestans* (Klug, 1835), which occupied household units among 12 states of Brazil and was considered the main vector involved in *T. cruzi* transmission in Brazil^11^ ^,^ ^12^. After the first standardized chemical-based control actions were implemented by the National Chagas Disease Control Program in 1975 and by the integrated initiatives of the Southern Cone of Latin America to eliminate *T. infestans* in 1991, a sustained decrease in domestic populations of vectors was observed^13^ ^-^ ^15^. In 2006, Brazil received certification of interruption of Chagas disease transmission by *T. infestans* from the Pan American Health Organization (PAHO) and the World Health Organization (WHO)^16^. However, many regions of Brazil, such as the state of Bahia, still have several species of native vectors that can transmit *T. cruzi* to humans and domestic animals.

Pirajá da Silva initially described the existence of triatomines in the state of Bahia in 1911, shortly after *T. cruzi* was described by Carlos Chagas^17^. Pirajá da Silva identified *Conorhinus megistus* triatomines - syn. *Panstrongylus megistus* (Burmeister, 1835) - in the city of Mata de São João, near Salvador, the capital city of Bahia. Pirajá da Silva also identified triatomines among the cities of Feira de Santana, Candeias, São Francisco do Conde, and Salvador^18^. Almost 40 years after Pirajá's initial research description, Chagas disease was recognized as a serious health problem in the state of Bahia, where triatomines were captured simultaneously to the record of autochthonous cases of Chagas heart disease in Salvador^19^. *P. megistus* and *Triatoma rubrofasciata* (De Geer, 1773) naturally infected by *T. cruzi* were found at the historic center of Salvador city, associated with human cases, which motivated a chemical control campaign^20^. Nevertheless, some foci of *P. megistus* maintained *T. cruzi* transmission to families in the neighborhoods of Salvador^20^. In the early 1970s, more than 600 specimens of *P. megistus* and *T. rubrofasciata* were examined in Salvador, of which 16% were infected with *T. cruzi* ^21^. During this period, efforts were made to identify and better understand the ecology of triatomines in Bahia, and 18 triatomine species were cataloged^22^. 

National campaigns focused on active vector surveillance for the identification of household infestation areas in Brazil and strategies of triatomine vector-control using chemical insecticides (BHC and pyrethroids). The 1983 Brazilian campaign was executed by the Superintendence of Public Health Campaigns (SUCAM) of Brazil’s Health Ministry. During this period, new triatomines were recorded, and new species were described^22^.

Recent epidemiological studies indicate that Bahia had a high prevalence of human *T. cruzi* infection (0.77% to 2.22%) compared to the Northeast region of Brazil (0.69% to 0.88%) between 1987 and 1994^15^. Data between 2008 and 2017 indicate that Chagas disease mortality rates in the state of Bahia (3.8 to 4.8 deaths/100,000 habitants) are the highest among the northeastern states and the fourth highest among all Brazilian states. Moreover, two deaths were registered in children younger than one year old, indicating acute cases and risk of domestic *T. cruzi* transmission^27^.

Currently, 26 triatomine species have been recognized in Bahia^25^ ^-^ ^26^. Several factors can modify the spatial distribution and abundance of synanthropic triatomines. To understand the changes in *T. cruzi* vector occurrence, the use of retrospective epidemiological and entomological data has become a relevant strategy. In this study, we describe the relative abundance and proportional spatial distribution of synanthropic triatomines at the municipal level before (1957 to 1971) and after (2006 to 2019) the vector-control program implementation in the state of Bahia, which is one of the states with the greatest diversity of triatomines and highest rates of epidemiological information related to Chagas disease in northeastern Brazil.

## METHODS

**FIGURE 1:**
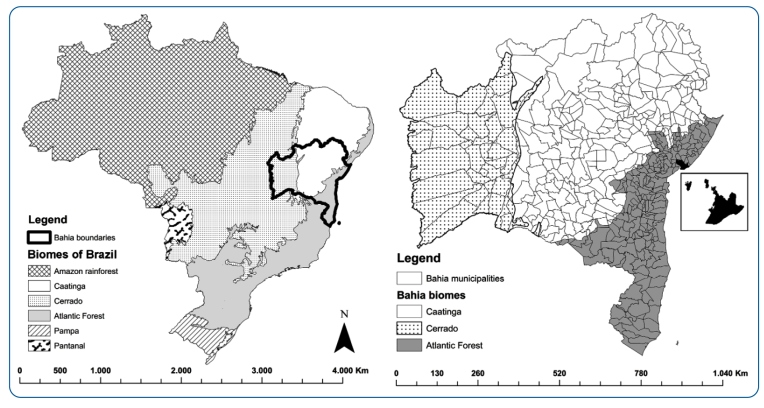
Study area. Biomes of Brazil and geolocation of Bahia **(a)**. Municipalities of Bahia **(b)**; the inset shows the city of Salvador (in detail).

The state of Bahia has 417 municipalities and is located in the southern region of northeastern Brazil, bordering eight other Brazilian states and the Atlantic Ocean in the east (Figure 1). Descriptive analyses of both the spatial and temporal distribution of the synanthropic triatomine species data from Bahia was performed during two periods: (A) from 1957 to 1971, and (B) from 2006 to 2019. Data regarding period A present information on the Chagas disease vector surveillance program of Bahia between 1957 and 1971^22^, which represents the period before the standardized implementation of systematic vector surveillance programs in Brazil^27^. 

Information regarding period B was obtained from the following state government databases after the certification of interruption of Chagas disease transmission by *T. infestans*: Epidemiologic Surveillance team of the State of Bahia’s Health Service (SESAB), Epidemiological Surveillance Office (DIVEP), and Central Public Health Laboratory of Bahia (LACEN/BA) ^29^.

Entomological data were obtained in three different ways: (a) notification of triatomines (surveillance and community participation) performed by the population itself by taking suspicious insects to health agents; (b) notification attendance (active surveillance) performed by the health agents of each municipality, dependent on (a); and (c) active search (active surveillance), in which vectors were searched among household units around the area, regardless of any notification performed by the population. 

To compare *T. cruzi* vector species information from both periods of the study, we evaluated: (1) the relative abundance, calculated as the proportion of each triatomine species divided by the total number of triatomines for each period; and (2) proportional spatial distribution, calculated as the proportion of municipalities that recorded a triatomine species divided by the number of sampled municipalities.

Spreadsheets were used to collect the following data: species, municipality, date, geographic information system (GIS) coordinates, sampling environment (intradomestic, peridomestic), and data reference. Graphs and descriptive statistics were computed using SPSS 24. In the absence of specific GIS information, the coordinates of the city's headquarters from the Brazilian Institute of Geography and Statistics (IBGE) were used^30^. Data processing was performed using the ArcGis® Software 10.5^31^. 

## RESULTS

In this study, information regarding 315 municipalities and 215597 triatomines collected during the two periods was evaluated, among which 35588 were from period A (1957-1971) and 180020 were from period B (2006-2019). During period A, data were gathered from 202/290 (71%) municipalities^22^, and in period B, 258/417 (61.8%) municipalities were evaluated. Regarding the number of triatomine species, 18 and 21 species were sampled during periods A and B, respectively, and 16 of them were sampled during both periods (Table 1). 

**TABLE 1:** Synanthropic triatomine species from the state of Bahia, Brazil, recorded between 1957-1971 and between 2006-2019.

Triatominae species	Presence		Specimens				Municipalities			
	A (1957-1971)	B (2006-2019)	A (n)	A (%)	B (n)	B (%)	A (n)	A (%)	B (n)	B (%)
*Cavernicola pilosa* Barber, 1937	*	*	**0**	-	**0**	-	**0**	-	**0**	-
*Panstrongylus lenti* Galvão & Palma, 1968;	*	*	**0**	-	**0**	-	**0**	-	**0**	-
*Panstrongylus lutzi* (Neiva & Pinto, 1923);	**X**	**X**	**62**	0.17	**369**	0.20	**11**	5.45	**66**	25.58
*Panstrongylus megistus* (Burmeister, 1835);	**X**	**X**	**22032**	61.93	**1842**	1.02	**122**	60.40	**38**	14.73
*Pantrongylus diasi* Pinto & Lent, 1946;	**X**	**X**	**17**	0.05	**6**	0.00	**5**	2.48	**1**	0.39
*Pantrongylus geniculatus* (Latreille, 1811);	**X**	**X**	**29**	0.08	**155**	0.09	**8**	3.96	**51**	19.77
*Parabelminus yurupucu* Lent & Wygodzinsky, 1979	*	*	**0**	-	**0**	-	**0**	-	**0**	-
*Psammolestes tertius* Lent & Jurberg, 1965;	**X**	**X**	**836**	2.35	**39**	0.02	**8**	3.96	**2**	0.78
*Rhodnius domesticus* Neiva & Pinto, 1923;	*	*	**0**	-	**0**	-	**0**	-	**0**	-
*Rhodnius nasutus* Stål, 1859		*	**0**	-	**16**	0.01	**0**	-	**0**	-
*Rhodnus neglectus* Lent, 1954;	**X**	**X**	**1**	0.00	**100**	0.06	**1**	0.50	**21**	8.14
*Triatoma bahiensis* Sherlock & Serafim, 1967;	**X**		**5**	0.01	**0**	-	**1**	0.50	**0**	-
*Triatoma brasiliensis* Neiva, 1911;	**X**	**X**	**1405**	3.95	**11054**	6.14	**18**	8.91	**92**	35.66
*Triatoma costalimai* Verano & Galvão, 1958;	**X**	**X**	**4**	0.01	**2**	0.00	**1**	0.50	**1**	0.39
*Triatoma infestans* (Klug, 1834);	**X**	**X**	**1310**	3.68	**763**	0.42	**26**	12.87	**7**	2.71
*Triatoma juazeirensis* Costa & Felix, 2007;		**X**	**0**	-	**225**	0.12	**0**	-	**8**	3.10
*Triatoma lenti* Sherlock & Serafim, 1967;	**X**	**X**	**56**	0.16	**226**	0.13	**3**	1.49	**2**	0.78
*Triatoma melanica* Costa, Argolo & Felix, 2006;		**X**	**0**	-	**19**	0.01	**0**	-	**1**	0.39
*Triatoma melanocephala* Neiva & Pinto, 1923;	**X**	**X**	**68**	0.19	**233**	0.13	**16**	7.92	**35**	13.57
*Triatoma pessoai* Sherlock & Serafim, 1967;	X**		**52**	0.15	**0**	-	**2**	0.99	**0**	-
*Triatoma petrocchiae* Pinto & Barreto, 1925;	**X**	**X**	**16**	0.04	**1**	0.00	**1**	0.50	**1**	0.39
*Triatoma pseudomaculata* Corrêa & Espínola, 1964;	**X**	**X**	**894**	2.51	**16717**	9.29	**40**	19.80	**174**	67.44
*Triatoma rubrofasciata* (De Geer, 1773);	**X**	**X**	**474**	1.33	**6**	0.00	**2**	0.99	**2**	0.78
*Triatoma sherlocki* Papa, Jurberg, Carcavallo, Cerqueira & Barata, 2002		X***	**0**	-	**323**	0.18	**0**	-	**1**	0.39
*Triatoma sordida* (Stål, 1859);	**X**	**X**	**8314**	23.37	**146901**	81.60	**75**	37.13	**157**	60.85
*Triatoma tibiamaculata* (Pinto, 1926);	**X**	**X**	**2**	0.01	**985**	0.55	**2**	0.99	**18**	6.98
*Triatoma vitticeps* (Stål, 1859);		**X**	**0**	-	**38**	0.02	**0**	-	**2**	0.78
*Triatoma brasiliensis* spp*#*	**X**	**X**	**1534**	4.31	**11848**	6.58	**22**	10.89	**94**	36.43
**TOTAL**	-	-	**35577**	100.00	**180020**	100.00	**202**	100.00	**258**	100.00

*Species have already been recorded in Bahia during another period. **Species today is considered synonymous with *T. lenti.* ***Captured by health agents in a wild environment. *#Triatoma brasiliensis* complex.

**FIGURE 2:**
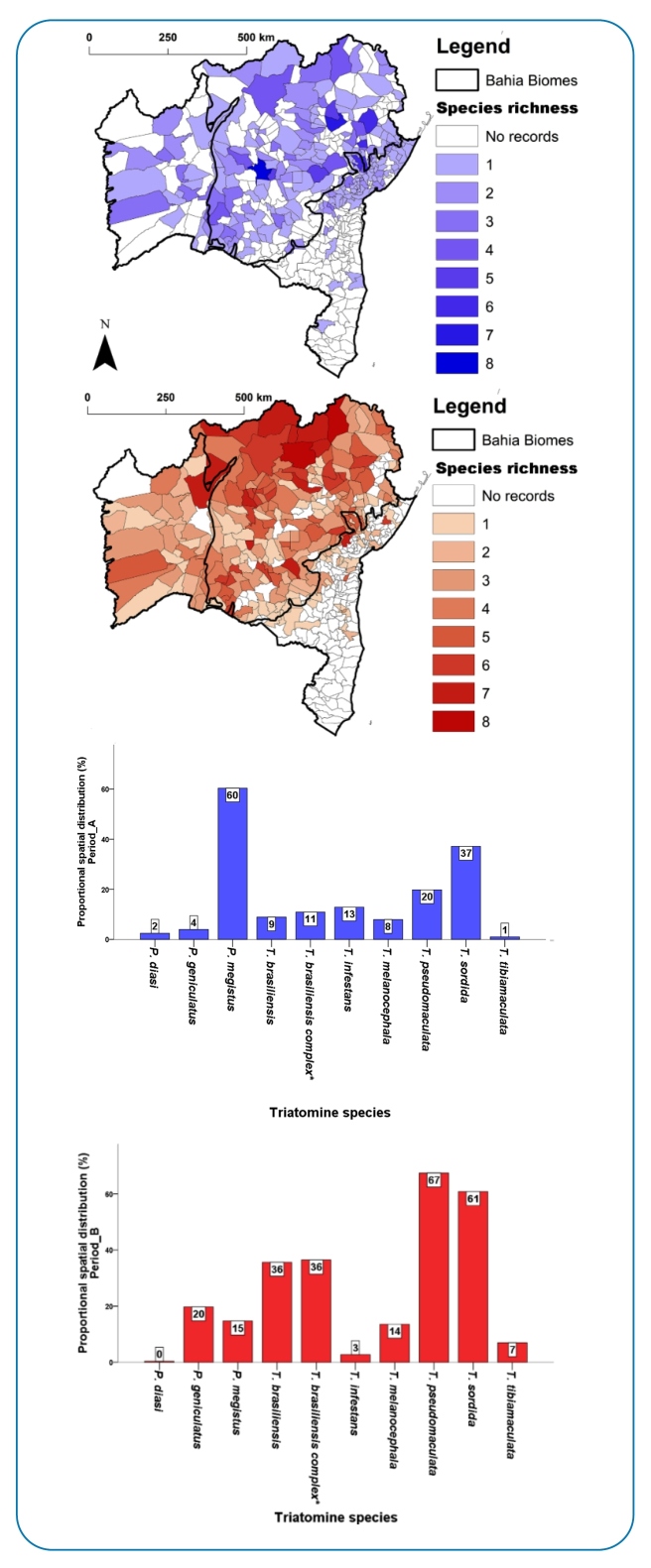
Municipalities with the occurrence of triatomines and species richness in the state of Bahia during periods A (1957-1971), in blue, and B (2006-2019), in red, and the spatial distribution of the synanthropic triatomines. Legend: Color gradients represent the species richness at each period of the study. “X” axis shows the name of the triatomine species evaluated, while “Y” axis shows the proportional (%) spatial distribution of them.

**FIGURE 3:**
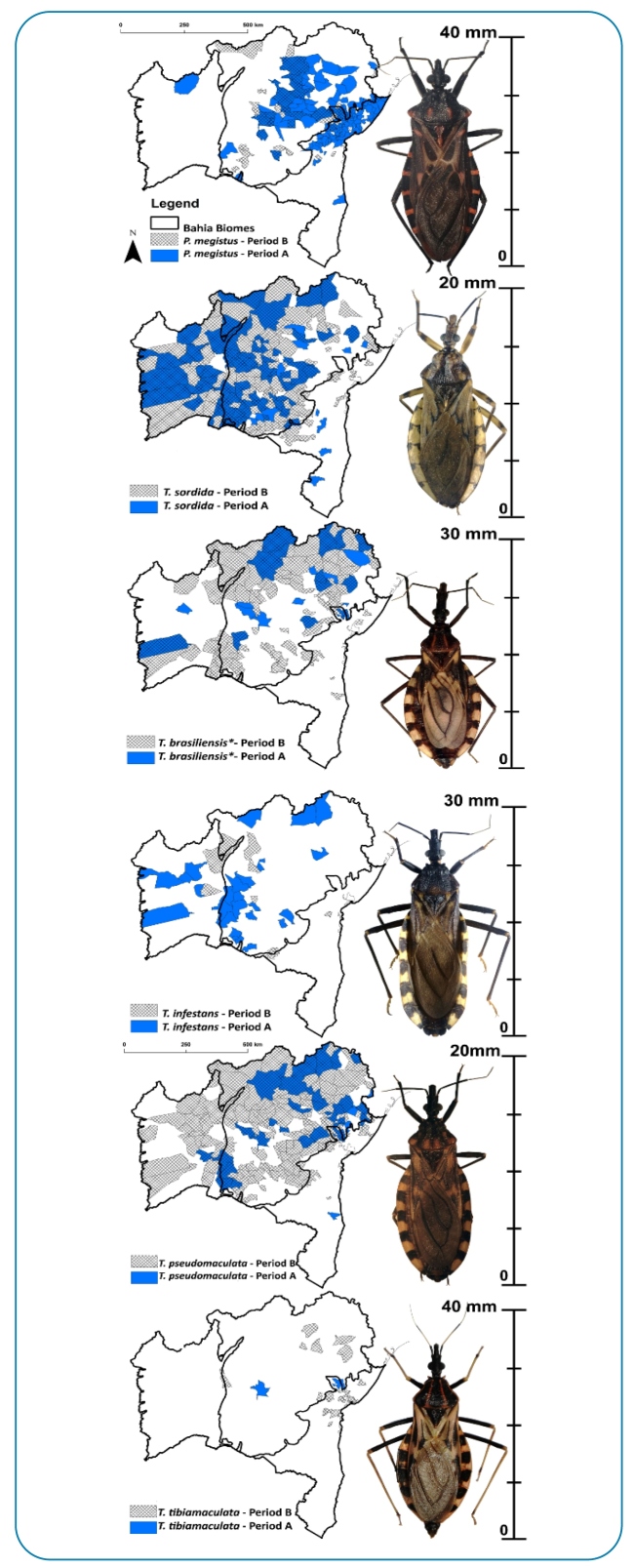
Geographic distribution of *Panstrongylus megistus*, *Triatoma infestans*, *T. pseudomaculata*, T. sordida, *T. brasiliensis species complex in the municipalities of the state of Bahia during periods A (1957-1971), in blue, and B (2006-2019), in crosshatch.

Figure 2 shows the spatial distribution of triatomines in the municipalities during periods A and B. During period A, *P. megistus* was present in 122 (60.4%) of the sampled municipalities, followed by *T. sordida* (37.1%), *T. pseudomaculata* (19.8%), and *T. infestans* (12.8%). During period B, *T. pseudomaculata* was widely distributed, being recorded in 176 of 258 (68.2%) sampled municipalities, followed by *T. sordida* (61.6%), and *T. brasiliensis* (36.4%) (Figure 3).

In period A, *P. megistus* was the most abundant species (n=22032), representing 61.9% of triatomines, followed by *T. sordida* (23.3%), *T. brasiliensis* species complex (4.3%), and *T. infestans* (3.6%). Conversely, in period B, *T. sordida* represented 81.6% of all collected triatomines, followed by *T. pseudomaculata* (9.2%) and *T. brasiliensis* species complex (6.3%).

By analyzing the spatial distribution of the main triatomine species before and after *T. cruzi* vector-control actions, we observed a reduction in the municipal occurrence of synanthropic populations of *T. infestans* and *P. megistus. T. infestans* was no longer detected in some municipalities in the west (e.g., Santa Maria Vitória, Barreiras) and north (e.g., Juazeiro, Curaçá) of Bahia (Figure 3) and had been first detected among other municipalities in the Caatinga (e.g., Itaguaçu da Bahia, Novo Horizonte) and Atlantic Forest biomes (e.g., Tremedal and Presidente Tancredo Neves). During period B, *T. infestans* was found only at residual foci and was last recorded in 2015 in the municipality of Novo Horizonte (Figure 3).

During period B, we observed a higher occurrence of *T. sordida* in western Bahia, where the Cerrado biome predominates, in south-central Bahia, and in some municipalities of Recôncavo Baiano, eastern Bahia. However, we observed a remarkable change in the spatial distribution of *T. pseudomaculata*, which expanded its area of occurrence among western municipalities (Cerrado biome) and the central region (Caatinga biome) of Bahia (Figure 3).

A comparison of the total number of individuals (n) and the relative abundance (%) of species in periods A and B showed a reduction for *P. megistus* (A: n=22032, 61.9%; B: n=1804, 1.0%) and *T. infestans* (A: n=1310, 3.6%; B: n=763, 0.4%), as well as an increase in the relative abundance of *T. sordida* (A: n=8314, 23.3%, B: n=146901, 81.6%) and *T. pseudomaculata* (A: n=894, 2.5%, B: n=16717, 9.2%). During period B, most triatomines were captured in peridomestic habitats, with a predominance of *T. sordida* (Table 2).

**TABLE 2:** Synanthropic triatomines collected in the state of Bahia, Brazil, by species and collection environment between 2006 and 2019.

Species	Intradomestic		Peridomestic		Not informed		Total	
	n	%	n	%	N	%	N	%
*Panstrongylus diasi*	6	0.04	0	0	0	0	6	0
*P. geniculatus*	64	0.41	43	0.03	48	1.01	155	0.09
*P. lutzi*	186	1.18	103	0.06	80	1.68	369	0.21
*P. megistus*	292	1.86	1435	0.9	115	2.41	1842	1.03
*Psammolestes tertius*	0	0	14	0.01	25	0.52	39	0.02
*Rhodnius nasutus*	4	0.03	0	0	12	0.25	16	0.01
*R. neglectus*	22	0.14	66	0.04	12	0.25	100	0.06
*Triatoma brasiliensis*	4281	27.23	6639	4.17	134	2.81	11054	6.15
*T. costalimai*	2	0.01	0	0	0	0	2	0
*T. infestans*	104	0.66	642	0.4	17	0.36	763	0.42
*T. juazeirensis*	141	0.9	42	0.03	42	0.88	225	0.13
*T. lenti*	19	0.12	197	0.12	10	0.21	226	0.13
*T. melanica*	0	0	19	0.01	0	0	19	0.01
*T. melanocephala*	32	0.2	4	0	197	4.13	233	0.13
*T. petrocchiae*	1	0.01	0	0	0	0	1	0
*T. pseudomaculata*	2348	14.94	13632	8.56	737	15.45	16717	9.3
*T. rubrofasciata*	4	0.03	0	0	2	0.04	6	0
*T. sordida*	7755	49.34	135865	85.34	3281	68.8	146901	81.75
*T. tibiamaculata*	456	2.9	500	0.31	29	0.61	985	0.55
*T. vitticeps*	2	0.01	8	0.01	28	0.59	38	0.02
**TOTAL**	**15719**	**100%**	**159209**	**100%**	**4769**	**100%**	**179697**	**100%**

Source: Health department of the state of Bahia (SESAB). Legend: N: absolute number of samples; Mun: Number of municipalities. %: percentage per column.

## DISCUSSION

This study indicated changes in the spatial distribution and relative abundance of synanthropic triatomines in the state of Bahia, Northeast Brazil, before and after 44 years of structured control actions of *T. cruzi* vectors, initiated in 1975. Our results indicate a clear reduction in the occurrence of *P. megistus* and *T. infestans* (last record in 2015) and an increase in the relative abundance and geographical distribution of *T. sordida* and *T. pseudomaculata* after the Brazilian vector-control program.

*Panstrongylus megistus* was the predominant species between 1957 and 1971; it was found in 60% of municipalities, with a relative abundance of 62% of collected triatomines^22^. Its proportional spatial distribution has been greatly reduced in the state of Bahia, especially in the areas of Recôncavo Baiano and the metropolitan region of Salvador. *P. megistus* spatial distribution has also been reduced in other Brazilian states^11^ ^,^ ^36^ ^-^ ^38^. Three hypotheses could explain this reduction in the metropolitan region of Salvador: (a) chemical control was successfully performed over four decades, resulting in the elimination of domestic populations of *P. megistus*; (b) intense urbanization in these municipalities resulted in deforestation and fragmentation of the Atlantic Forest biome areas, which is the natural habitat of this species^22^; and (c) housing improvement, with progressive depletion of adobe houses^39^ ^,^ ^40^, a favorable environment for *P. megistus* colonization. *P. megistus* is a native Brazilian species with a wide spatial distribution and high epidemiological and entomological relevance due to the high rates of *T. cruzi* infection and its proximity to human dwellings, as it can colonize intra-and/or peridomicile areas. In 2021, *P. megistus* foci were described in the metropolitan region of São Paulo, with *T. cruzi*-infected triatomines associated with marsupials, revealing the importance of continued surveillance of synanthropic *P. megistus* ^46^.

During period A, *T. infestans* were found in 12.8% of the sampled municipalities and represented 3.7% of the sampled triatomines. During period B, *T. infestans* was identified in only seven municipalities with residual colonies^34^ ^-^ ^35^. Bahia was the last Brazilian state to receive PAHO certification, possibly because of specific *T. infestans* identification errors and the appearance of new records of this species in the study area^32^. The elimination of *T. infestans* in several municipalities in western and central Bahia can be explained by the *T. infestans* elimination plan. This plan was intensified in 2004 and included spraying households with insecticides, followed by research and capture of triatomines, surveying approximately 500000 households^26^. However, residual colonies of this species have been detected in a few municipalities^28^ ^,^ ^29^, requiring constant monitoring to eliminate residual foci from the state of Bahia. In addition to the chemical control performed in Bahia since 1975, intensified in 1991, which aimed to eliminate *T. infestans* ^11^ ^,^ ^15^, other social actions were implemented by the federal government. These included the growth acceleration program, intended to improve housing quality, with the replacement of mud houses with brick houses and health education actions on Chagas disease. This may also have influenced the reduction in household colonization by *T. infestans* and other household species^39^ ^,^ ^40^ ^.^

Although successful in controlling *T. infestans*, several native species have been recorded in a large number of municipalities. They were captured in households and frequently colonized peri domestic areas. Among them, some were infected by *T. cruzi* and many of them fed on domestic animals and human blood^42^. In period A, *T. sordida* was recorded in 37% of the municipalities, representing 23% of the collected triatomines, while *T. pseudomaculata* was recorded in 19% of the municipalities, with a relative abundance of 2.5% of the gathered triatomines^23^. In period B, *T. sordida* was recorded in 60% of the municipalities, representing approximately 81% of the triatomines. *T. sordida* is the most common species in different regions of Bahia. Similarly, *T. pseudomaculata* was identified in the largest number of municipalities during period B (67.4%). Systematic vector-control actions had a low impact on the spatial distribution of *T. sordida* and *T. pseudomaculata* in Bahia. There was a higher occurrence of *T. sordida* in western and south-central Bahia, where there are areas of Cerrado, which is the original biome of the species’ natural populations^1^ ^,^ ^2^ ^,^ ^25^. The highest occurrence of *T. pseudomaculata* in western and central Bahia followed the occurrence predictions of the species based on environmental variables^42^. The number of specimens of *T. sordida* and *T. pseudomaculata* exceeded 90% of all triatomines collected in the state between 2006 and 2019.

We observed a higher occurrence of *T. brasiliensis* and similar species in Bahia, thus expanding the observations of Ribeiro-Jr *et. al.* ^41^. Before systematized control actions, the *T. brasiliensis* species complex was registered in 10% of the municipalities of Bahia, and between 2006 and 2019, at least 94 municipalities (36%) registered this species. In the last few years, other species of the *T. brasiliensis* complex have been described in the state of Bahia, including *T. juazeirensis*, *T. melanica*, *T. sherlocki*, and *T. petrocchiae* ^43^. Among these species, *T. juazeirensis* is noteworthy because it was predominantly collected inside household units. Other species, such as *T. tibiamaculata*, *P. geniculatus*, *T. melanocephala*, and *P. lutzi*, were intrusively detected inside the houses, mainly adult specimens.

There was a significant difference in the occurrence of *T. tibiamaculata* between the two periods. In period A, it was recorded in approximately 1% of the municipalities with a relative abundance of 0.01%^23^. In period B, it was recorded in 7% of the municipalities with a relative abundance of 0.55%. In recent decades, even though *T. tibiamaculata* has been naturally found inside nests of marsupials, rodents, and epiphytes in forests^1^ ^,^ ^22^, it has been recorded in peri domestic palms and inside households. Therefore, *T. cruzi-*infected *T. tibiamaculata* generates a transmission risk in Salvador, probably in the entire metropolitan region, and in the Atlantic Forest areas^44^.

Triatomine vectors of *T. cruzi* can be classified as native/non native, wild/non wild, domestic/peridomestic to enable the definition of effective vector-control strategies^45^. While domestic and peridomestic populations of species of triatomines are subject to spraying of chemical pesticides, which is the main strategy of vector control, native-wild species can persistently invade or colonize the household (peri-and intradomicile). Thus, this may represent a challenge for controlling *T. cruzi* transmission in the domestic environment.

This study has several limitations. Since the use of reference databases does not allow a broad analysis of data, it was impossible to obtain data on triatomines within habitats (intra-and peridomicile) in period A (1957-1971). The effects of passive dispersion and seasonal changes on vector behavior were impossible to measure. In addition, the database does not allow descriptive evaluations of nymph occurrence. Moreover, not all municipalities collected data regularly during the evaluated periods, and the health surveillance service classifies municipalities among the high, medium, and low risk of transmission, emphasizing that there is no obligation to conduct regular entomological research among low-risk municipalities based on the classification presented by Brazil’s Health Ministry^26^. Future studies reassessing risk classification at the municipal level are urgently needed for the redefinition of areas and risk of transmission. Further studies should analyze the situation of these areas to explain whether the absence of triatomines is due to the functioning of the service or biogeographic issues related to triatomines. In periods A and B, *T. brasiliensis* complex species were considered as one, and to avoid misinterpretations, data obtained in this study were evaluated at *stricto sensu* level^44^. In Bahia, nine species that are unlikely to be found within households^24^ ^,^ ^25^ ^,^ ^33^ were recorded by the surveillance services. These included *Belminus laportei*, *Eratyrus mucronatus*, *Microtriatoma trinidadensis*, *Panstrongylus tupynambai*, *P. lignarius*, *Rhodnius prolixus*, *Triatoma maculata*, *T. circummaculata,* and *T. rubrovaria*. To reduce taxonomic identification errors, a guide was developed^33^ and, more recently, a guidance manual for the surveillance of triatomines in Bahia, with identification keys, diagnosis, and distribution of vector species^26^.

This study describes changes in the triatomine fauna of Bahia between the analyzed periods more than 40 years after the implementation of the vector-control program in the state. During this period, we observed changes in the Chagas disease surveillance policies in Brazil due to decentralization. The centralized Brazilian control program was transferred to municipalities without corresponding training. The decentralization policy of the Chagas disease vector-control program aimed to bring the actions of the government closer to the communities, but true engagement by local communities has not been achieved. After decentralization, the efficacy of some municipalities in detecting triatomines has been reduced. We observed a reduction in the relative abundance and proportional spatial distribution of domestic and domesticated species of *T. cruzi* vectors *P. megistus* and *T. infestans,* respectively*.* In addition, we described an increase in the native species *T. sordida*, *T. pseudomaculata,* and *T. brasiliensis* species subcomplex and highlighted the important role of native triatomine species in *T. cruzi* transmission in the domiciliary environment. The observed changes in *T. cruzi* main vectors between the evaluated periods demonstrate the importance of reinforcing entomological surveillance actions. Furthermore, promoting and disseminating community-based scientific knowledge and health education actions on Chagas disease at a local scale will help mitigate surveillance challenges and control native triatomines.

### Ethics

The procedures followed the ethical standards of the Research Ethics Committee of the Gonçalo Moniz Institute (IGM - FIOCRUZ, Bahia, Brazil). The consent form was waived since the analysis was performed using a database. However, to provide full anonymity to participants, no personal identification data were used. The research did not cause any physical, psychological, moral, intellectual, social, cultural, or religious risks to the residents or animals in the study areas. In addition, this study did not include endangered or protected species.
